# Exploring health service preparation for the COVID-19 crisis utilizing simulation-based activities in a Norwegian hospital: a qualitative case study

**DOI:** 10.1186/s12913-022-07826-5

**Published:** 2022-04-26

**Authors:** Une Elisabeth Stømer, Peter Dieckmann, Thomas Laudal, Kristi Bjørnes Skeie, Sigrun Anna Qvindesland, Hege Langli Ersdal

**Affiliations:** 1 Research Department, Stavanger University Hospital, Stavanger, Norway; 2 Department of Quality and Health Technology, Faculty of Health Sciences, University of Stavanger, Stavanger, Norway; 3 Copenhagen Academy for Medical Education and Simulation (CAMES), Herlev Hospital, Capital Region of Denmark, Denmark; 4 Department of Public Health, Copenhagen University, Copenhagen, Denmark; 5 Stavanger Business School, University of Stavanger, Stavanger, Norway; 6 Critical Care and Anaesthesiology Research Group, Stavanger University Hospital, Stavanger, Norway

**Keywords:** COVID-19 pandemic, Simulation-based activities, Hospital leaders, Simulation facilitators, Institutional learning

## Abstract

**Introduction:**

The first wave of the COVID-19 pandemic caused stress in healthcare organizations worldwide. Hospitals and healthcare institutions had to reorganize their services to meet the demands of the crisis. In this case study, we focus on the role of simulation as part of the pandemic preparations in a large hospital in Norway. The aim of this study is to explore hospital leaders' and simulation facilitators' expectations of, and experiences of utilizing simulation-based activities in the preparations for the COVID-19 pandemic.

**Methods:**

This is a qualitative case study utilizing semi-structured in-depth interviews with hospital leaders and simulation facilitators in one large hospital in Norway. The data were sorted under three predefined research topics and further analyzed by inductive, thematic analysis according to Braun and Clarke within these pre-defined topics.

**Results:**

Eleven members of the hospital leadership and simulation facilitators were included in the study. We identified four themes explaining why COVID-19 related simulation-based activities were initiated, and perceived consequences of the activities; 1) a multifaceted method like simulation fitted a multifaceted crisis, 2) a well-established culture for simulation in the hospital was crucial for scaling up simulation-based activities during the crisis, 3) potential risks were outweighed by the advantages of utilizing simulation-based activities, and finally 4) hospital leaders and simulation facilitators retrospectively assessed the use of simulation-based activities as appropriate to prepare for a pandemic crisis.

**Conclusions:**

The hospital leadership’s decision to utilize simulation-based activities in preparing for the COVID-19 crisis may be explained by many factors. First, it seems that many years of experience with systematic use of simulation-based activities within the hospital can explain the trust in simulation as a valuable tool that were easy to reach. Second, both hospital leaders and simulation facilitators saw simulation as a unique tool for the optimization of the COVID-19 response due to the wide applicability of the method. According to hospital leaders and simulation facilitators, simulation-based activities revealed critical gaps in training and competence levels, treatment protocols, patient logistics, and environmental shortcomings that were acted upon, suggesting that institutional learning took place.

**Supplementary Information:**

The online version contains supplementary material available at 10.1186/s12913-022-07826-5.

## Introduction

The initial outbreak of the COVID-19 pandemic was an enormous challenge to hospitals around the world. In many countries, hospitals experienced that the number of patients exceeded their capacity. The risk of spreading the virus required changes in clinical routines, treatment protocols, patient logistics, and physical environments. During the first wave of the COVID-19 pandemic, there was limited knowledge about the course of the disease, the treatment possibilities, and the contagiousness of the virus. A study investigating preparedness assessment in a Middle East hospital revealed several safety threats due to the pandemic such as constantly shifting routines in the treatment protocols, lack of confidence in infection control, as well as overall panic in an unknown situation [1]. Videos and news from Italian hospitals revealed chaotic conditions which put significant psychologic stress on healthcare professionals, and hospitals around the world were told to prepare for worst-case scenarios [2, 3].

One way to prepare for a situation that requires new ways of working is scaling up simulation-based activities (SBA), which was a chosen strategy by the case hospital in this study. SBA are recognized as an excellent method to increase competence in healthcare professionals and has shown to improve patient outcomes in numerous specialties such as obstetrics, trauma care, pediatrics, and emergencies due to better communication between team members, optimized workflow, and better routines for transferring patients to appropriate ward levels [4–12].

Several recent studies worldwide have investigated hospitals’ utilization of SBA during the COVID-19 pandemic. For example, So et al. described how a hospital in Hong Kong established a COVID-19 training task force to run multidisciplinary endotracheal intubation training [13]. In the US, a large community teaching hospital used in-situ simulation to revise their code blue protocol to meet COVID-19 challenges, and train staff [14]. Other studies have also investigated hospitals’ experiences from utilizing SBA during the COVID-19 pandemic, with varying focus; improving care and identifying safety issues [15], training hospital staff and system learning [16], preparing teams and environment for covid-19 patients [17], describing the use of simulation in covid-19 [18], observing safety threats and test possible solutions [19], process optimization [20], testing PPE in resuscitation [21] and devices as well as predicting resources and the contagiousness of the virus [22–27].

An alternative strategy in a pandemic would be to cancel all SBA for fear of infection of healthcare workers in an uncertain situation. A potential benefit of the latter move would be to shift simulation facilitators with clinical backgrounds to clinical work and thereby increase their capacity. However, this would reduce capacity and ability to run SBA for staff, and probably require re-entry training of educational staff.

### Simulation and simulation-based activities

Simulation is defined as “an educational technique that replaces or amplifies real experiences with guided experiences that evoke or replicate substantial aspects of the real world in a fully interactive manner” [28]. SBA may be understood as “the entire set of actions and events from initiating to termination of an individual simulation event; in the learning setting, this is often considered to begin with the briefing and end with the debriefing” [29]. In-situ simulation, which means SBA taking place at the workplace of those participating, is particularly suitable for difficult work environments, due to space constraints and the advantages of training in the environment where the skills are to be used. For example, an ambulance, a small aircraft, a dentist’s chair, a catheterization lab – all of these settings present physical characteristics that are relevant for task management, but which are difficult to re-create [29]. In-situ SBA is recognized as appropriate to probe systems and the workflow, safety risks may thereby be identified, and corrections can be made without harming patients or hospital staff in real situations [30–33].

SBA is also recognized as a method to build confidence and well-being at an individual level by increasing healthcare professionals’ self-confidence in working in teams, and in performing technical skills for example donning and doffing personal protection equipment (PPE) [34–36].

To improve teamwork capabilities, one needs to practice skills that require interactions to succeed which can be accomplished by teamwork simulations [16, 30, 35, 37].

SBA has become more common in hospitals over the last decades [38] but is not yet systematically implemented as an educational method in all Norwegian hospitals [39].

Trust, or confidence, among participants, is essential in the successful execution of simulation in hospitals [40, 41]. This is also critical to maintaining an absorptive capacity among the employees, which in turn ensures that the lessons learned are fully explored and acted on in the organization [42]. Studies have found that SBA, and in particular debriefings, may produce the trust required for learning [4, 41].

### Institutional learning

Schön points out that learning in an institutional setting depends on the practitioners’ reflection [43]. The debriefing part, happening after the simulation, enables those involved to reflect on the simulated experience. By jointly reconstructing the event – possibly seeing inconsistencies and misunderstandings, by finding explanations for different dynamics, and by analyzing the consequences of actions, a deeper insight into ones’ own dynamics and the dynamic in the group can become more obvious. When the discussion is then also related to recognized theoretical concepts, a deeper understanding and a more efficient and safe way of acting can be identified and implanted [44]. A challenge is to enlarge the learning effect from those directly involved in the session to the whole organization and current simulation practice is focusing on this process of distribution of expertise. We assume that SBA, performed with good quality, may contribute with learning-opportunities not only to the individuals taking part in in the SBA, but also to institutional learning. For example, allowing members of the organization to act as “learning agents” and disseminate knowledge to other members of the organization, and to learn from the experiences of others [45].

Dieckmann et al. describe the potential contribution of simulation during the COVID-19 pandemic in three areas: creating new learning opportunities, optimizing workflows, and dealing with the emotional stress of employees [46].

Summing up simulation-based activities and learning in hospitals.

The advantages of using large-scale SBA in hospitals are numerous and beyond individual and team learning. SBAs have the potential to identify learning needs and risk factors both at the individual and systemic levels. SBA also provides methods to test new ways of working without harming patients or staff and reducing personal stress among employees. In the end, the advantages may contribute to improved patient outcomes. Even though many studies have been focused on the role of SBA in preparing for the COVID-19 pandemic, none of these studies focused on the experiences of the responsible staff for initiating and executing SBA in such demanding circumstances. This study contributes to the existing body of related studies by focusing on the experiences of the responsible staff at the case hospital.

Three research topics were predefined:

1. What did hospital leaders and simulation facilitators expect of SBA in the preparation for the pandemic?
2. What drivers and barriers for SBA were experienced during the pandemic?
3. How did SBA contribute during the first wave of COVID-19, according to hospital leaders and simulation facilitators?

## Methods

This is a qualitative case study utilizing individual in-depth interviews with members of the case hospital’s leadership and simulation facilitators.

### Case hospital

The case hospital serves approximately 370 000 people in the western part of Norway and has more than 8000 employees. SBA were well established in the organization and involve staff from various departments, backgrounds, and levels of experience based on many years of collaboration in numerous projects. At the beginning of March 2020, the hospital went on yellow alert, which enabled the hospital to expedite quick decision-making processes and implement measures deemed necessary to meet the pandemic crisis. Senior management at the hospital immediately requested the establishment of a collaborative task force to plan and run SBA to help during Covid-19. Of particular importance was (1) Focus attention on the health and safety of the employees, and (2) Ensure satisfactory patient management. The task force’s mission was to complement and assist the hospital’s emergency procedures to cope with the COVID-19 crisis. The collaborative units were comprised of clinical simulation facilitators at the hospital, resources at the hospital’s Dept. of Education, an affiliated local simulation center and, the members of the regional simulation network. In addition, representatives from the Infectious Control Disease Unit staff members from the Dept. of Education were delegated to support these efforts.

### Ethical aspects and consent to participate

The study was submitted to and approved by the Data Protection Officer at Stavanger University Hospital (ID-1405), which is the appropriate ethical review board for health service research according to the national legislation. The participants signed informed consent before the interviews were conducted. The participants were informed that participation was voluntary and that they could withdraw from the project at any point.

### Simulation-based activities

During the period of investigation (March 8’th – April 28’th 2021), 36 team simulations relevant to COVID-19 were performed involving 693 employees according to the records from the simulation task force. These were already ongoing in-situ team simulations pre-Covid-19 and involved the following emergency teams:

- Cardiac arrest team simulation
- Stroke team simulation
- Paediatric-crisis team simulation
- Peri-natal team simulation
- Trauma team simulation
- Critically ill adult team simulation
- Deterioration of septic patient in bed-unit

Following the taskforce mandate, and collaboration with the clinical simulation leads responsible for these team simulations, all in-situ team simulations continued but the focus in all scenarios was to suspect patients of having Covid-19. Participants represented the actual members of the teams in focus including paramedics, physicians of relevant specialties, nurses of relevant specialties, bioengineers, porters, radiographers, and midwives typical to their multi-professional groups. There was a shortage of PPE supplies during this first wave so hand-made facemasks, normal latex gloves, re-usable protection glasses, and washable protective frocks were used. Efforts were made to make debriefings as time-efficient as possible and participants were told to spread out spatially to heed infection control advice. The primary learning objectives were to help staff training on health and safety issues, including infection control, and practice satisfactory COVID-19 patient treatment given the team in focus. Except for the cardiac arrest team training which used manikins designed for training advanced cardiac arrest, all simulated patients in the scenarios were healthcare workers on call and prepared for their roles or simulation task force members. All facilitators had taken the Level 1 Train-The-Trainer courses following the EUSim (Courses – EuSim). In most cases, they had long simulation experience and were clinicians who worked with the teams in question as part of their regular work. In some cases, the simulation task force participated as co-facilitators, operators of simulated monitors, or simulated patients, as well as assisting with planning and collecting learning points. The infection control staff were invited to attend the simulations as professional advisors and observers. They attended when possible but were in high demand across the hospital for pandemic-associated queries and work.

### Participants in this study

We used the organizational map of the employees, e-mails, and simulation activity documentation from the period to identify relevant participants.

### Inclusion criteria

Members of the hospital’s leadership and simulation facilitators who were involved in either making decisions about utilizing SBA or involved in organizing and performing SBA during the period of investigation.

Eleven members of the management staff (hospital leaders) and eight simulation facilitators were invited to participate, seven hospital leaders (three medical doctors and four nurses) and four simulation facilitators (three medical doctors and one nurse) agreed. The median age of the participants was 49 years (range 37–61), and seven (63%) were identified as women.

### Data collection/Interviews

Three researchers (UES, TL, and KBS) conducted the interviews which took place in a private room at the case hospital or at the participants’ current workplace during October 2020. All interviews were audiotaped and transcribed verbatim, by the same researchers, within six weeks. The interviews lasted from 27 to 58 min (mean, 47 min). The semi-structured interview guide was sent to the participants in advance to prepare them since the period of investigation had been 6 months earlier. UES, TL and PD performed the data analysis. The researchers did not participate in the planning or running of the SBA during the period of investigation and had different professional backgrounds to each other.

The interview guide is included as Additional file 1.

### Data analysis

NVivo 16 software for qualitative analysis was used to collate the dataset into the three predefined research topics. Further, thematic analysis (TA) was used to systematically organize the data into a structured format and to identify themes and sub-themes of significance. We followed the six phases of TA as described by Braun and Clarke [47], see Table 1.

**Table 1:** Six phases of Thematic Analysis by Braun and Clarke, and our analysis activity

Braun and Clarkes’ six phases of thematic analysis with descriptions		Our analysis activity
1. Familiarize with the data	Transcribing the data, reading and re-reading the data, noting down initial ideas	After transcription, each researcher read the interview transcripts several times
**2.** Generating initial codes	Coding interesting features of the data in a systematic fashion across the whole dataset, collecting data relevant to each code	The researchers noted initial codes during the phase of familiarizing with the dataset
**3.** Searching for themes	Collating codes into potential themes, gathering all data relevant to each potential theme	The researchers collated the different codes into the three predefined research questions by the use of the NVIVO 16 software system for qualitative analysis. In a final reading, the researchers identified sub-themes within each of the four main topics
**4.** Reviewing themes	Checking if the themes work in relation to the coded extracts (phase 1) and the entire dataset (phase 2), generating a thematic ‘map’ of the analysis	The researchers discussed the initial codes and recoded some of these as part of this discussion. The theme structure was then consolidated
**5.** Defining and naming themes	Ongoing analysis to refine the specifics of each theme, and the overall story the analysis tells, generating clear definitions and names for each theme	Researchers compared notes and agreed on the final sample of quotes illustrating the themes
**6.** Producing the report	The final opportunity for analysis. Selection of vivid, compelling extract examples, final analysis of selected extracts, relating back of the analysis to the research question and literature, producing a scholarly report of the analysis	The themes and subthemes were interpreted in light of the three research questions

NVivo = software solution for organizing and analyzing qualitative data. Source of the two first columns of the table: Braun and Clarke [47]

## Results

We identified four themes relevant to our research topics:

1. a multifaceted method like SBA fitted a multifaceted crisis,
2. a well-established culture for simulation in the hospital was crucial for scaling up SBA during the crisis,
3. potential risks were outweighed by the advantages of utilizing SBA, and finally
4. hospital leaders and simulation facilitators retrospectively assessed the use of SBA as an appropriate way to prepare for a pandemic crisis.

A multifaceted method like simulation fitted a multifaceted crisis.

The participants described great diversity in aims and expectations of using SBA to prepare for the pandemic. The sub-themes describe various expectations from hospital leaders and simulation facilitators such as promoting simulation as a method, trying out new ways of working and organizing the work, as well as identifying the employees’ learning needs, and reducing personal stress, Table 2.

**Table 2:** A multifaceted method like simulation-based activities fitted a multifaceted crisis

Research topic	Citation	Sub-theme	Theme
**Expectations**	As I remember it now, I had high expectations and I also thought that this will be a huge opportunity to test out what we have talked about for so long. Namely to use a day to find out where the training needs are (for simulation-based training) (ID-1)	Educational opportunity: Promote simulation as an educational method	A multifaceted method including SBA fitted a multifaceted crisis
	… it is an incredibly much better way to learn than just sending someone an email or an e-learning course or a sheet to read things through. That you actually have to do things is so much more effective to learn than just using **one** sense. (ID-2)		
	That is, what space we should use in the emergency room. How to move from the emergency room to the operating room. How we were to function in the X-ray, i.e. in the CT room and who was to be in and where they should stand (ID- 10)	System focus: System probing and testing out new ways of working	
	And they also implemented new equipment and new ways of working, such as intubating. During a normal intubation, you stand with your face 10 approx. from the uvula. But then (due to COVID-19) they started intubate with a video laryngoscope…. and then it's a good way to try it out in simulation (ID-7)		
	..but if we first take infection control equipment then it became quite early clear that there are many who need to practice. (ID-2)	Personal focus: Identifying learning needs in employees	

ID- number refers to different participants. *SBA* Simulation-based activities

The first citation in Table 2 describes the pandemic as a “giant opportunity” to test out SBA as a tool to face a crisis. By quickly identifying learning needs, and thereby creating targeted learning opportunities with relevant simulation activities/scenarios, the organization could optimize the effort in training based on the employees' needs instead of shooting wide without knowing what the learning needs were. The expectations linked to SBA reflect the different focus areas as described in Dieckmann et al.: educational focus, system focus, and personal focus.

A well-established culture for simulation was crucial for scaling up simulation-based activities during the crisis.

Even if the aims of using SBA were multifaceted among the participants, there seemed to be a consensus that it was a natural choice of method for both hospital leaders and simulation facilitators. This was by many linked to the extensive experience with SBA at the case hospital before the COVID-19 crisis occurred. Both top-down anchoring of simulation as a method in the organization as well as bottom-up enthusiasm among the employees and simulation facilitators were of significance during the crisis, Table 3.

**Table 3:** A well-established culture for simulation was crucial for scaling up simulation-based activities

Research topic:	Citation	Sub-theme	Theme
**Driving factors**	We have focused on what is it we can use simulation to help with (which settings?) (ID- 5)	Long experience with simulation	A well-established culture for simulation was crucial for scaling up SBA during the crisis
	I think we had an advantage because our basic system (for using simulation) was in place.. we did not need to establish a new basic system. (ID- 7)		
	We are so integrated with simulation training at the XXX hospital that this is what we use when something is difficult. We have even used simulation to learn how to avoid conflicts. It is integrated as part of a toolbox that you can use for all sorts of weird things. (ID-8)	Top-down anchoring of simulation/ managers see the benefit of simulation-based activities	
	… XXX got XXX sewing-team to sew face masks and they used reusable infection coats to practice with… eh so they managed to respond to the challenges (lack of PPE) there and then (ID-6)	Pro-active and creative simulation enthusiasts in the wards/departments	

ID-number refers to different participants. *SBA* Simulation-based activities

Potential risks were outweighed by the advantages of utilizing SBA.

Both managers and facilitators experienced concerns due to the SBA. The concerns were about spreading the virus by simulating in teams, overusing PPE, and fear of wrong learning, as described in Table 4.

**Table 4:** Potential risks were outweighed by the advantages of utilizing simulation-based activities

Research topic:	Citation	Sub-theme	Theme
**Barriers/concerns**	The simulations in small rooms with 15–20 persons involved… I do not know who made those decisions … but we stopped unnecessary observers and interns….. So it was the current team remaining in the room. And we would have all been together the next day if it had been a real situation, no matter who took that balancing about benefit up against infection control risk and outcome I do not know. (ID-6)	Risk of infection	Potential risks were outweighed by the advantages of utilizing SBA
	we actually thought about that and then eh… then it was said that well we have to take that risk because it is so important (to perform simulation-based activities) (ID-7)		
	… It is demanding and a big risk (to perform simulation-based activities) but gives an outcome that makes it worth it (ID-6)		
	..there was a real discussion right there… very challenging because there was a precarious lack of equipment right? So when we said yes to using infection coats for simulation eh… when you then knew that you then may not have infection coats for when I need it, was a trade-off we had to take (ID- 1)	Lack of PPE	
	We got new staff. But what I really was concerned about was the danger of infection. Here we suddenly got a bunch of people who did not know infection control who were to teach infection control to others, something they could not and I was very stressed for that reason. (ID-3)	Fear of wrong learning	

ID-number refers to different participants. *SBA* Simulation-based activities

None of the participants described any heavy discussions, disagreements, or strong counterarguments against SBA during the research period. It seems to be an agreement that the benefits were higher than the risks. Questions about cost-effectiveness were answered by counter questions of the legitimacy of asking for the monetary cost:

*….this is a kind of impossible discussion I would say because that’s what it is .. one thing is the cost finances and such, but what you have to put in the other end is the effect. What does it cost to provide good treatment? And what does it cost if you give poor treatment? (ID-8)*

Hospital leaders and simulation facilitators retrospectively assess the use of simulation-based activities as appropriate to prepare for a pandemic crisis.

Participants described many different contributions from the SBA. According to some, the SBA led to adjustments of written protocols, reorganizing the physical environment and, increased acceptance of simulation as a pedagogical method, Table 5.

**Table 5:** Hospital leaders and simulation facilitators retrospectively assess the use of simulation-based activities as appropriate

Research topic:	Citation	Sub-theme	Theme
**Experienced contributions of simulation**	We have learned that recruiting and training and caring for healthcare professionals in a pandemic situation like this is incredibly important—or you can have as much equipment and facilities as you want, but it breaks down. So perhaps to have an even greater focus on how the simulation-based training can take care of the psychosocial issues.. (ID-8)	Personal focus: Reduced stress and increased well-being for employees	Hospital leaders and simulation facilitators retrospectively assess the use of SBA as appropriate to prepare for a pandemic crisis
	yes we have gathered all the teams because in the beginning everyone sat and wrote about their procedures for the teams. And one team had one kind of infection control rule, the other had different infection control rules and the third had yet other infection control rules. Then an anesthesia nurse came back from quarantine and he also reads the procedures, he also says that they are about the same things but there are different things. Here you must gather. We also changed the procedures. So all the things there have come into place now. (ID-6)	System focus: Revealed several risk factors in the hospital that were improved Consolidated written procedures and making them more coherent	
	There has been a lot of talk about it afterwards. We really saw the utility of simulation during the pandemic. Of course, I was about to say that if the strategy was not rooted before, then it is now. At SimRåd (the hospital simulation advisory board) which was recent, it was like… then people were much more involved in the management team in terms of understanding how to use simulation… (ID-4)	Organizational focus: Increased the credibility for simulation as a method both internally and externally	
	I do not think we would be able to reach so many if you were to collect personnel to a kind of lecture… So I have now learned that it can be an incredibly potent tool to ensure people such a basic knowledge that is absolutely essential In such a situation as this is. (ID-8)	Educational focus: Simulation appears to be an effective pedagogical method	

ID- number refers to different participants. *SBA* Simulation-based activities

Although the facilitators and managers seem to retrospectively assess SBA as an appropriate strategic move to prepare for the pandemic, there is also natural scepticism that the modest number of COVID-19 patients that were admitted to the case hospital did not represent a stress test for the feasibility, as described by the following citations:

- Because we never got the big wave. we are just preparing for the big wave (ID-7).
- We never got to stress test the system (ID-11)

The results show that the expectations towards SBA were not confined to individual learning opportunities. They included improvements of systemic features and identified also learning needs and competence gaps. A mentioned element in support of SBA during the pandemic was the extensive experience with simulation over many years, while mentioned barriers were the risk of infecting colleagues and the lack of PPE. Finally, SBA seemed to contribute to a well-targeted and effective improvement of competencies according to the hospital leaders and simulation facilitators.

## Discussion

We aimed to explore and explain hospital leaders' and simulation facilitators’ experiences with SBA in preparation for the COVID-19 pandemic in a large Norwegian hospital. Our results show that a well-established culture for simulation throughout the organization was a key factor for the motivation when scaling up SBA. Both hospital leaders and simulation facilitators experienced that SBA contributed in ways that may be considered as institutional learning.

### Expectations to simulation-based activities during the first wave of COVID-19

Videos and news from Italian hospitals revealed chaotic situations that were terrifying to watch. However, these chaotic situations most likely contributed as a motivating factor for preparing healthcare institutions worldwide for the pandemic. The case hospital had some time to prepare for the pandemic, even so, decisions had to be made quickly. The yellow alert status allowed for rapid decisions, and the case hospital quickly chose to scale up SBA. Scaling up SBA was debated in the healthcare sector due to the nature of a pandemic. Our results reveal that SBA was a highly valued tool that was used for several different purposes in the case hospital, including any difficulties/problems that needed to be solved. The trust in simulation as an effective tool for solving problems seemed to be a reason for the slightly controversial choice of strategy. Another reason might have been the established structure for SBA in the case hospital. All participants described simulation as a well-known method for training in the hospital. Further, the internal structures were already established and systematic team simulations were performed regularly. The established structure for SBA made it easy to reach for the hospital leaders and easy to organize for the simulation facilitators.

### Perceived contributions of simulation-based activities during the initial wave of COVID-19

Our results indicate skepticism between different task force members, re: Table 4: Potential risks were outweighed by the advantages of utilizing simulation-based activities. The fear of wrong learning in some participants suggests that a shared mental model and continual communication between members and different contributing professions in the task force could have been clearer. The lack of clear communication may have been attributed to the stressful and uncertain nature of the pandemic that brought supporting units together. The supporting units had not collaborated on a systematic basis pre-COVID-19 did not know each other's exact competencies and may not have established collaborative trust.

According to the results, the role of SBA in preparing for COVID-19 was to increase existing simulation activities including both individual and systemic focus on the risk of spreading the virus. Facilitators collaborated across internal organizational boundaries to quickly develop courses around practical skills (like donning and doffing PPE) and organized logistics around the production of a substantial number of training opportunities. SBA helped find effective clinical set-ups and in making existing approaches more efficient. The SBA also helped to activate more human resources by initiating new and facilitating existing collaborations i.e., the decision about inviting the simulation task force to assist the hospital in the preparations.

SBA also extended beyond intensifying and scaling up existing activities in the COVID-19 context. Simulations and debriefings were used to explore systemic issues. Individual challenges that became clear during the sessions were investigated for implications beyond the individual. If there were indications for wider implications, they were reported back to the organization through feedback loops in which the simulation facilitators could inform relevant leadership. These formal feedback loops would have been absent without organized SBA. According to Nonaka and Takeuchi, externalization of learning into the organization happens whenever knowledge obtained in debriefings is encoded into guidelines, written procedures, or made part of the organization’s formal structure in some way [48]. Our results, therefore, suggest that SBA led to institutional learning that most likely not would have occurred without SBA.

There were also specific simulations, which aimed at finding organizational, technical, procedural bottlenecks and best practices. This clear system focus is not related to individual learning but aims at different levels of system/institutional learning. Finally, simulation extended not only into the organization. SBA also extended into the personal focus. Scenarios and debriefings became a vehicle to set the light on the personal and emotional strain of workload, insecurity about the disease, fear of being infected, and other implications of COVID-19 [42].

Considering that teamwork simulation tends to increase the trust and confidence of the participants, SBA was not only able to address the unique competence needs relying on individual levels, but also to stimulate a work environment that is supportive of institutional learning. Thus, our findings support Dieckmann et al.'s postulation that SBA have great potential to help mitigate the negative effects of the COVID-19 crisis [46].

### Strengths and limitations

This study has both strengths and limitations. The strengths were a multi-professional team of researchers conducting the interviews together, ensuring similar interviews situations. The multi-professionalism in the researcher team led to fruitful discussions around data analysis and the theoretical approach. Further, none of the researchers who were involved in data collection and analysis were active in supporting the case hospital SBA during the period of investigation and thereby did not have any interest in boasting the results, which increases the trustworthiness of the research. The data from the interviews were rich and the researchers agreed on saturation which also increases the trustworthiness of the study. A limitation would be the single-center, case study design limiting generalization. However, the case hospital represented a unique approach towards the pandemic in Norway which might be of great interest to other healthcare organizations to learn from in future situations. Another limitation was the five-month, time gap between the period of interest, and from the period of interviews. The time gap might have weakened the memory of the participants, however; it may also have contributed to more reflection about the situation and a better overview of the total situation. The low number of participants might be considered as a limitation related to the representativeness of the data. However, it became clear during the interviews that a level of saturation occurred, meaning that the last interviews did not reveal many new insights. The qualitative design of the study per se limits the possibility of extrapolation of the findings, but not the transferability to similar situations.

### Implications for practice

To our knowledge, the case hospital was the only hospital in the country which utilized SBA as a strategy to prepare for the pandemic. Other hospitals in Norway chose to pause SBA in the initial phase of the pandemic due to the uncertain circumstances; hence, our results are of great value to both the Norwegian hospitals and the nation's healthcare system. The management and the simulation facilitators' experiences reveal a throughout trust in SBA as a pedagogical method that made it easy to scale up and adapt to pandemic preparations. The well-established culture for SBA can inspire other healthcare institutions to establish such a culture in their organizations. It would seem important that to activate educational services in a crisis, including SBA, these services should be established, tried out, and trusted with competent leaders and facilitators able to dynamically adjust activities to meet new challenges. A lesson learned from this study is when rapidly creating a task force to assist a hospital, all collaborative units must establish reciprocal expectations to contributions. These expectations should include how to collaborate and maintain constructive communication lines for feedback and adjustments to provide the best training opportunities to front-line staff and truly benefit mutually by collaboration.

## Conclusion

The leadership and simulation facilitators in the case hospital expected-, and experienced SBA to be a feasible strategy to prepare the organization for the COVID-19 pandemic. The multifaceted potentials of SBA fit the new requirements of Covid-19 where health workers needed to find new ways of working in addition to identifying individual and systemic limitations. The positive attitudes toward SBA were a crucial driver and seemed to be related to the long and well-established culture of simulation in the case hospital. The experiences from the case hospital leaders and simulation facilitators indicate that SBA contributed beyond individual learning and system probing, it also contributed to organizational learning which might be of inspiration for other healthcare institutions. However, the findings from this case study need to be supported by additional research due to the modest number of Covid-19 patients.

## Supplementary Information

**Additional file 1.**

## Data Availability

The dataset supporting the conclusions of this article are included within the article and its additional file.

## Abbreviations

PPE: Personal Protection Equipment
SBA: Simulation-based activities

## References

[1] Sharara-Chami R, Sabouneh R, Zeineddine R, Banat R, Fayad J, Lakissian Z (2020). In situ simulation: an essential tool for safe preparedness for the COVID-19 pandemic. Simul Healthc.

[2] Ramsay S. Coronavirus: Italy's hardest-hit city wants you to see how COVID-19 is affecting its hospitals Sky News: Sky News; 2020 [Available from: https://news.sky.com/story/coronavirus-they-call-it-the-apocalypse-inside-italys-hardest-hit-hospital-11960597.

[3] Bostock B. Horrowing video from a hospital at the center of Italy's coronavirus outbreak shows doctors overwhelmed by critical patients. Insider: Insider; 2020 [Available from: https://www.businessinsider.com/video-tour-coronavirus-icu-ward-bergamo-italy-worst-apocalyptic-2020-3?r=US&IR=T.

[4] Theilen U, Fraser L, Jones P, Leonard P, Simpson D (2017). Regular in-situ simulation training of paediatric medical emergency team leads to sustained improvements in hospital response to deteriorating patients, improved outcomes in intensive care and financial savings. Resuscitation.

[5] Capella J, Smith S, Philp A, Putnam T, Gilbert C, Fry W (2010). Teamwork training improves the clinical care of trauma patients. J Surg Educ.

[6] Murphy M, McCloughen A, Curtis K (2019). The impact of simulated multidisciplinary trauma team training on team performance: a qualitative study. Australas Emerg Care.

[7] Wisborg T, Brattebø G, Brinchmann-Hansen Å, Uggen PE, Hansen KS (2008). Effects of nationwide training of multiprofessional trauma teams in norwegian hospitals. J Trauma Acute Care Surg.

[8] Park C, Grant J, Dumas RP, Dultz L, Shoultz TH, Scott DJ (2020). Does simulation work? Monthly trauma simulation and procedural training are associated with decreased time to intervention. J Trauma Acute Care Surg.

[9] Sharara-Chami R, Lakissian Z, Farha R, Tamim H, Batley N (2020). In-Situ simulation for enhancing teamwork in the emergency department. Am J Emerg Med.

[10] Ajmi SC, Advani R, Fjetland L, Kurz KD, Lindner T, Qvindesland SA (2019). Reducing door-to-needle times in stroke thrombolysis to 13 min through protocol revision and simulation training: a quality improvement project in a Norwegian stroke centre. BMJ Qual Saf.

[11] Steinemann S, Berg B, Skinner A, DiTulio A, Anzelon K, Terada K (2011). In situ, multidisciplinary, simulation-based teamwork training improves early trauma care. J Surg Educ.

[12] Brattebø G, Ersdal HL, Wisborg T. Simulation-based team training works. Tidsskr Nor Laegeforen. 2019;139(18). Norwegian, English. 10.4045/tidsskr.19.0565. 10.4045/tidsskr.19.0565 31823569

[13] So EHK, Chia NH, Ng GWY, Chan OPK, Yuen SL, Lung DC, et al. Multidisciplinary simulation training for endotracheal intubation during COVID-19 in one Hong Kong regional hospital: strengthening of existing procedures and preparedness. BMJ Simul Techn Enhanc Learn. 2021:bmjstel-2020–000766. 10.1136/bmjstel-2020-000766. Epub 2021 May 25. 10.1136/bmjstel-2020-000766 PMC8154296 35520980

[14] Youssef FA, Patel M, Park H, Patel JV, Leo J, Tanios MA (2021). Protected code blue: using in situ simulation to develop a protected code blue and modify staff training protocol—experience in a large community teaching hospital during the COVID-19 pandemic. BMJ Open Quality.

[15] Kurz MW, Ospel JM, DaehliKurz K, Goyal M (2020). Improving stroke care in times of the COVID-19 pandemic through simulation: practice your protocols!. Stroke.

[16] Wenlock RD, Arnold A, Patel H, Kirtchuk D (2020). Low-fidelity simulation of medical emergency and cardiac arrest responses in a suspected COVID-19 patient - an interim report. Clin Med (Lond).

[17] Muhsen WS, Marshall-Roberts R. Simulation-guided preparations for the management of suspected or confirmed COVID-19 cases in the obstetric emergency theater. J Matern Fetal Neonatal Med. 2022;35(9):1801–4. 10.1080/14767058.2020.1765333. Epub 2020 May 19. 10.1080/14767058.2020.1765333 32429714

[18] Chaplin T, McColl T, Petrosoniak A, Hall AK (2020). “Building the plane as you fly”: simulation during the COVID-19 pandemic. Canadian Journal of Emergency Medicine.

[19] Choi GY, Wan WT, Chan AK, Tong SK, Poon ST, Joynt GM (2020). Preparedness for COVID-19: in situ simulation to enhance infection control systems in the intensive care unit. Br J Anaesth.

[20] Muret-Wagstaff SL, Collins JS, Mashman DL, Patel SG, Pettorini K, Rosen SA (2020). In situ simulation enables operating room agility in the COVID-19 pandemic. Ann Surg.

[21] Malysz M, Dabrowski M, Böttiger BW, Smereka J, Kulak K, Szarpak A (2020). Resuscitation of the patient with suspected/confirmed COVID-19 when wearing personal protective equipment: a randomized multicenter crossover simulation trial. Cardiol J.

[22] Begley J, Lavery K, Nickson C, Brewster D (2020). The aerosol box for intubation in coronavirus disease 2019 patients: an in-situ simulation crossover study. Anaesthesia.

[23] Gardiner C, Veall J, Lockhart S (2020). The use of UV fluorescent powder for COVID-19 airway management simulation training. Anaesthesia.

[24] Sharma D, Rubel KE, Ye MJ, Shipchandler TZ, Wu AW, Higgins TS, et al. Cadaveric simulation of endoscopic endonasal procedures: analysis of droplet splatter patterns during the COVID-19 pandemic. Otolaryngol Head Neck Surg. 2020;163(1):145–50. 10.1177/0194599820929274 PMC7240308 32423283

[25] Shojaee S, Pourhoseingholi MA, Ashtari S, Vahedian-Azimi A, Asadzadeh-Aghdaei H, Zali MR (2020). Predicting the mortality due to Covid-19 by the next month for Italy, Iran and South Korea; a simulation study. Gastroenterol Hepatol Bed Bench.

[26] Kristiansen IS, Burger EA, Blasio BF. Covid-19: Simulation models for epidemics. Tidsskr Nor Laegeforen. 2020;140(6). English, Norwegian. 10.4045/tidsskr.20.0225. 10.4045/tidsskr.20.0225 32321234

[27] Alban A, Chick SE, Dongelmans DA, Vlaar AP, Sent D (2020). ICU capacity management during the COVID-19 pandemic using a process simulation. Intensive Care Med.

[28] Gaba DM (2004). The future vision of simulation in health care. Qual Saf Health Care.

[29] Lioce L. Healthcare Simulation Dictionary, 2020 [Available from: https://www.ahrq.gov/sites/default/files/wysiwyg/professionals/quality-patient-safety/patient-safety-resources/research/simulation_dictionary/sim-dictionary.pdf.

[30] Gaba DM (2007). The future vision of simulation in healthcare. Simul Healthc.

[31] Sollid SJM, Dieckman P, Aase K, Soreide E, Ringsted C, Ostergaard D (2019). Five topics health care simulation can address to improve patient safety: results from a consensus process. J Patient Saf.

[32] Kjaergaard-Andersen G, Ibsgaard P, Paltved C, Irene Jensen H (2021). An in situ simulation program: a quantitative and qualitative prospective study identifying latent safety threats and examining participant experiences. Int J Qual Health Care.

[33] Colman N, Dalpiaz A, Walter S, Chambers MS, Hebbar KB (2020). SAFEE: a debriefing tool to identify latent conditions in simulation-based hospital design testing. Adv Simul.

[34] Reedy GB (2015). Using cognitive load theory to inform simulation design and practice. Clin Simul Nurs.

[35] Abulebda K, Ahmed RA, Auerbach MA, Bona AM, Falvo LE, Hughes PG (2020). National preparedness survey of pediatric intensive care units with simulation centers during the coronavirus pandemic. World J Crit Care Med.

[36] Wong AH, Ahmed RA, Ray JM, Khan H, Hughes PG, McCoy CE (2021). Supporting the quadruple aim using simulation and human factors during COVID-19 care. Am J Med Qual.

[37] Lie SA, Wong LT, Chee M, Chong SY (2020). Process-oriented in situ simulation is a valuable tool to rapidly ensure operating room preparedness for COVID-19 outbreak. Simul Healthc.

[38] Okuda Y, Bond W, Bonfante G, McLaughlin S, Spillane L, Wang E (2008). National growth in simulation training within emergency medicine residency programs, 2003–2008. Acad Emerg Med.

[39] (Norwegian governement document): Helse- og omsorgsdepartementet (2019). Meld. St. 7 (2019–2020) Nasjonal helse- og sykehusplan 2020–2023. https://www.regjeringen.no/no/dokumenter/meld.-st.-7-20192020/id2678667/.

[40] Wilford A, Doyle TJ (2006). Integrating simulation training into the nursing curriculum. Br J Nurs.

[41] Gilfoyle E, Ng E, Gottesman RD, Grant VJ, Cheng A. Comprehensive Healthcare Simulation. Pediatrics. 2016;43-54. ISSN: 2366-4479 , 2366-4487; ISBN: 3-319-24185-0 , 3-319-24187-7. 10.1007/978-3-319-24187-6_4.

[42] Cohen WM, Levinthal DA. Absorptive capacity: A new perspective on learning and innovation. Administrative science quarterly. 1990. p. 128–52.

[43] Schon D (1983). donald schon (schön): learning, reflection and change. Accessed April.

[44] Husebø SE, O’Regan S, Nestel D. Reflective practice and its role in simulation. Clin Simul Nurs. 2015;11(8):368–75.

[45] Argyris C, Schön DA. Organizational learning: A theory of action perspective. Reis. 1997(77/78):345–8.

[46] Dieckmann P, Torgeirsen K, Qvindesland SA, Thomas L, Bushell V, Langli EH (2020). The use of simulation to prepare and improve responses to infectious disease outbreaks like COVID-19: practical tips and resources from Norway, Denmark, and the UK. Adv Simul (Lond).

[47] Braun V, Clarke V (2006). Using thematic analysis in psychology. Qual Res Psychol.

[48] Nonaka I, Takeuchi H. The knowledge-creating company: How Japanese companies create the dynamics of innovation (Vol. 105). OUP USA. 1995.

