# Supplementary material for: Exploring health service preparation for the COVID-19 crisis utilizing simulation-based activities in a Norwegian hospital: a qualitative case study
Source: BMC Health Serv Res. 2022 Apr 26;22:563. doi: 10.1186/s12913-022-07826-5 (PMC9041286; doi:10.1186/s12913-022-07826-5)
Supplement: Supplementary file 1 — Additional file 1. [file 12913_2022_7826_MOESM1_ESM.docx]

**Interview guide – Hospital leaders**

1. For how long have you been employed at this hospital?
2. How would you describe your daily tasks at work from March – April 2020?
3. What kind of experience with simulation-based activities did you have at the hospital before the COVID-19 crisis?
4. Could you explain what role simulation-based activities played at the hospital before the COVID-19 crisis?
5. Are you familiar with any significant strategic choices made to meet the COVID-19 pandemic at this hospital?
6. What kind of expectations did you have of simulation-based activities as a method to cope with, and prepare for, the pandemic?
7. Imagine that you were to prepare for the COVID-19 crisis *without* simulation-based activities. How would you manage? Which pros and cons do you see for simulation-based activities and alternative strategies without simulation?
8. In your mind, why did the hospital use simulation-based activities to cope with the COVID-19 crisis?
9. What are your most important experiences after having to cope with COVID-19 this far?
10. Are you aware of practical changes at the hospital due to the simulation-based activities during the COVID-19 crisis?
11. Why do you believe the hospital used simulation-based activities to cope with the COVID-19 crisis?
12. What do you believe the hospital organization learned, or should have learned, from applying simulation-based activities during the COVID-19 crisis?
13. Is it your impression that this hospital stood out compared to other hospitals you know of when it comes to the use of simulation-based activities during the COVID-19 crisis?
14. Is there anything else you would like to add, related to these issues?

##### Interview guide – Simulation facilitators

1. For how long have you been employed at this hospital?
2. How would you describe your daily tasks at work from March – April 2020?
3. What experience with simulation-based activities did you have at the hospital before the COVID-19 crisis?
4. What is the importance, if any, of simulation-based activities in your position at the hospital?
5. What were your thoughts when it was decided that simulation-based activities were to be utilized to cope with the COVID-19 crisis at this hospital?
6. What kind of expectations did you have of simulation-based activities as a method to cope with the pandemic?
7. Imagine that you were to prepare for the COVID-19 crisis *without* simulation-based activities. How would you manage? Which pros and cons do you see for simulation-based activities and alternative strategies without simulation?
8. What are your most important experiences after having to cope with COVID-19 this far?
9. Are you aware of practical changes at the hospital due to the simulation-based activities during the COVID-19 crisis?
10. Why do you believe the hospital used simulation-based activities to cope with the COVID-19 crisis?
11. How significant were the simulation-based activities for coping with COVID-19 at this hospital? Can you give examples?
12. What do you believe the hospital organization learned, or should have learned, from applying simulation-based activities during the COVID-19 crisis?
13. Is there anything else you would like to add, related to these issues?
